# Intravenous Iron Dextran as a Component of Anemia Management in Chronic Kidney Disease: A Report of Safety and Efficacy

**DOI:** 10.1155/2013/703038

**Published:** 2013-03-18

**Authors:** Lenar Yessayan, Ankur Sandhu, Anatole Besarab, Alexy Yessayan, Stan Frinak, Gerard Zasuwa, Jerry Yee

**Affiliations:** ^1^Division of Nephrology and Hypertension, Henry Ford Hospital, 2799 West Grand Boulevard, Detroit, MI 48202, USA; ^2^St. Alexius Medical Center, 900 East Broadway Avenue, Bismarck, ND 58501, USA; ^3^Statberry.com, P.O. Box 371, Irvine, CA 92650, USA

## Abstract

*Objective*. We aimed to demonstrate safety and efficacy of intravenous (IV) low molecular weight iron dextran (LMWID) during treatment of anemic stage 3 and 4 chronic kidney disease (CKD) patients. Methods. Efficacy data was obtained by retrospective chart review of 150 consecutively enrolled patients. Patients were assigned per protocol to oral or IV iron, with IV iron given to those with lower iron stores and/or hemoglobin. Iron and darbepoetin were administered to achieve and maintain hemoglobin at 10–12 g/dL. Efficacy endpoints were mean hemoglobin and change in iron indices approximately 30 and 60 days after enrollment. Safety data was obtained by retrospective review of reported adverse drug events (ADEs) following 1699 infusions of LMWID (0.5–1.0 g). *Results*. Mean hemoglobin, iron saturation, and ferritin increased significantly from baseline to 60 days in patients assigned to LMWID (hemoglobin: 11.3 versus 9.4 g/dL; iron saturation: 24% versus 12.9%; ferritin: 294.7 versus 134.7 ng/mL; all *P*  values < 0.0001). Iron stores and hemoglobin were maintained in the group assigned to oral iron. Of 1699 iron dextran infusions, three ADEs occurred. *Conclusions*. Treatment of anemia in CKD stages 3 and 4 with LMWID and darbepoetin is efficacious. The serious ADE rate was 0.06% per infusion.

## 1. Introduction

Erythropoiesis is optimized in chronic kidney disease by treatment with iron and erythropoiesis-stimulating agents (ESAs). For end-stage renal disease patients on hemodialysis, intravenous (IV) iron is more efficacious than oral iron [1–3]. Potential advantages of IV iron include direct iron delivery to bone marrow and tissue stores, large-dose delivery, and elimination of frequent gastrointestinal side effects associated with oral iron treatment [4–8]. Among IV iron formulations, iron isomaltoside, ferric carboxymaltose, and the iron dextrans (IDs) can be administered in total dose infusion (TDI). Both ferric gluconate and iron sucrose can be safely administered as a bolus or short infusion at doses up to 250 mg and 300 mg, respectively. Higher doses of either drug as a bolus or short infusion have been associated with unpleasant vasoactive and gastrointestinal symptoms [9, 10]. However, accelerated regimens of high-dose IV iron sucrose (500 mg over 3 hours) have been demonstrated to be safe in several studies [11–13]. The safety and efficacy of ID in nondialysis chronic kidney disease (ND-CKD) is less well reported, especially for the low molecular weight iron dextran (LMWID) INFeD [14]. Imferon, Dexferrum, and INFeD are the IDs that have been used in the USA. The first two are high molecular weight iron dextrans (HMWIDs); the last is an LMWIDs. Imferon is no longer available; it was withdrawn by the original manufacturer, Fisons, based on economic decisions [15, 16]. These preparations are erroneously classified by some as a single class of product with similar side-effect profile. However, recently published literature revealed that LMWID has a different safety profile than HMWID. Several studies have shown that HMWID is associated with more adverse drug events (ADEs) than other iron preparations in the market [17–20]. The level and methods of ADE reporting vary between different studies. Some report the ADE rate per episode of ID administered, others per unit ID infusion or per patients treated. Moreover, there is no uniform definition of what constitutes a “serious” adverse event [21].

After recent concerns about the safety of ESAs and the current economic realities, the need for more effective anemia management strategies is of paramount importance. Incorporation of IV iron into the anemia treatment paradigm is needed to achieve the lowest effective ESA dose. At the Henry Ford Hospital Chronic Kidney Disease Clinic, LMWID in conjunction with ESA is used for ND-CKD anemia management using a computerized algorithm for dosing (see Section 2). ID is infused in accelerated fashion (1.5–2 hours) and in amounts considered to be TDI. This study reports the 2-month outcomes of hemoglobin (Hb), ferritin, and transferrin saturation (TSAT) in both IV and oral treatment groups from baseline. The cumulative incidence of ADEs related to ID in a cohort of 935 patients followed in ND-CKD is also reported.

## 2. Methods

### 2.1. Safety

This study was conducted in two parts. For the safety outcome, a retrospective chart review was done of all ND-CKD patients who received iron dextran (INFeD) between January 2001 and November 2005 in the outpatient CKD clinic at Henry Ford Hospital, Detroit, Michigan. The 936 ND-CKD subjects identified in the database each received either 0.5 or 1.0 g infusion by peripheral vein over 1.5–2.0 hours. The dosing of INFeD during this study period was at the discretion of the treating physician. None of our patients were premedicated with diphenhydramine. Safety endpoints were ADEs documented in the patients' electronic records. ADE rate were reported per episode of ID administered. All ADE presumed to be related to LMWID were also reported to the Food and Drug Administration (FDA) MedWatch. Serious ADEs were classified as cardiovascular collapse and anaphylactoid shock. Moderate ADEs were categorized as dyspnea, severe urticaria, chest discomfort, or neck/back spasms. Mild ADEs were classified as headache, dizziness, tachycardia, and hypertension in which the infusion was stopped but the patient subsequently completed the infusion [22].

### 2.2. Efficacy

**Figure 1:**
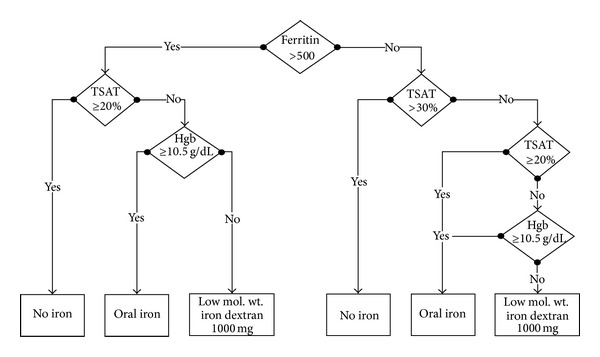
Computerized iron dosing protocol for CKD stages 3 and 4.

The efficacy outcomes were obtained from analysis of the data of patients enrolled in the clinic's computerized anemia management program (CAMP) between December 2005 and January 2007. CAMP is designed to treat anemia of CKD using darbepoetin alpha (DA) and iron treatment algorithms. After manual data input, the iron dosing algorithm prescribes no iron, oral iron, or 1 g of INFeD over 1.5–2 hours based on Hb, TSAT, and ferritin levels. At all times, iron “sufficiency” is to be achieved/maintained with oral or parenteral ID per the algorithm (Figure 1). ESA dosing is based on three separate protocols for first, second, and maintenance DA doses with a target to achieve and maintain Hb in the range of 10.0–12.0 g/dL. The first DA dose is based on the entry Hb. The second dose is based on the initial DA dose, initial Hb and current Hb. Maintenance DA doses are based on trend analysis of the most recent Hb, the last two DA doses and any increase or decrease in the last two doses.

All subjects were ≥18 years old, ND-CKD with an estimated glomerular filtration rate (GFR) of ≤60 mL/min, and fulfilled criteria for iron deficiency anemia per the CAMP protocol. For the 26-month period, 166 subjects were found in the database of CAMP who received and completed IV or oral iron treatment as prescribed by CAMP.

Sixteen patients were excluded because either their second evaluation day period exceeded 37 days from baseline or the third evaluation day exceeded 37 days from second evaluation day. Among the remaining 150 patients, 50 received LMWID and 100 received oral iron because their parameters never fulfilled the criteria to receive IV iron per CAMP. Oral iron was administered as Nephron FA. Each tablet contained 200 mg of ferrous fumarate (33% elemental iron), 40 mg of ascorbic acid, 1 mg folate, 75 mg of sodium docusate, and all B vitamins. Prior to being enrolled in CAMP, 32 of 50 patients assigned to IV iron had received DA in the past three months. In the oral iron group, 75 of 100 patients had received DA. No patient in either group had received IV iron in the past three months. Only 2 of 50 patients assigned to IV iron group needed a second dose of LMWID on second evaluation.

The primary efficacy endpoint was the mean Hb in each group. Secondary endpoints were iron indices (TSAT, ferritin) at *≈*30 and 60 days in each group as well as ESA dose requirements. Since the two groups by protocol design were assigned to different iron and ESA dosing, and dosing was adjusted to reach or maintain a target goal; we only compared mean differences between the two groups at the third clinic visit (*≈*60 days).

### 2.3. Statistical Analysis

Categorical variables are presented as frequencies and percentages. Continuous variables are presented using mean ± standard deviation. Comparisons of baseline characteristics between oral and IV iron groups were tested using two-sample *t*-tests for continuous variables, and chi-square and Fisher's exact tests for categorical variables. Intra-individual changes between time points for Hb, ferritin, TSAT, and DA were tested using Student's paired *t*-test. For all analyses, a *P* value < 0.05 was considered significant. Statistical analysis was performed using SAS software version 9.2 (SAS Institute, Inc., Cary, NC) and graphs were produced using STATA software version 10 (StataCorp LP, College Station, TX).

## 3. Results

### 3.1. Demographics

For the 150 patients represented in the efficacy analysis, baseline characteristics of both oral and IV iron groups are listed in Table 1. There was no statistical difference in age, race, or gender between frequencies of coronary artery disease, diabetes, and hypertension. However, the patients who received IV iron had a higher frequency of congestive heart failure. Baseline Hb, TSAT, and ferritin were lower in the IV group, as expected based on CAMP. For the 935 patients represented in the safety analysis, the mean age was 63.9 ± 15.0 years; 60% were women; 64% were African American, 32% white, and 4% either Asian or Hispanic.

### 3.2. Safety

Over the 6-year study period, 935 ND-CKD patients (488 stage 3 CKD and 447 stage 4 CKD) were treated with ID. A total of 1699 infusions (14 × 1 gram and 1685 × 0.5 gram doses) were administered over the study period (1713 0.5 g equivalents). Three ADEs were reported. Only one ADE was classified as serious. The overall ADE event rate was 0.175% per 0.5 gram dose equivalents and 0.177% per episode of IV iron infusion. The rate for serious ADE was less than 0.06%. All three ADEs occurred in women (mean age 70.3 years [range, 50–85 years]). All ADEs occurred with the first dose and within 30 minutes of ID administration. In these patients, a test dose did not predict the occurrence of a subsequent ADE. Of the three patients with ADEs, one experienced a severe reaction requiring 1 mg of epinephrine to reverse hypotension and bradycardia; one experienced a moderate reaction with dyspnea and responded to diphenhydramine; the third experienced a moderate reaction with a rash that responded to diphenhydramine and dexamethasone treatment. None of the three patients required hospitalization or Emergency Department evaluation. There were no fatalities noted from ID administration during the 6-year study period.

### 3.3. Efficacy

**Figure 2:**
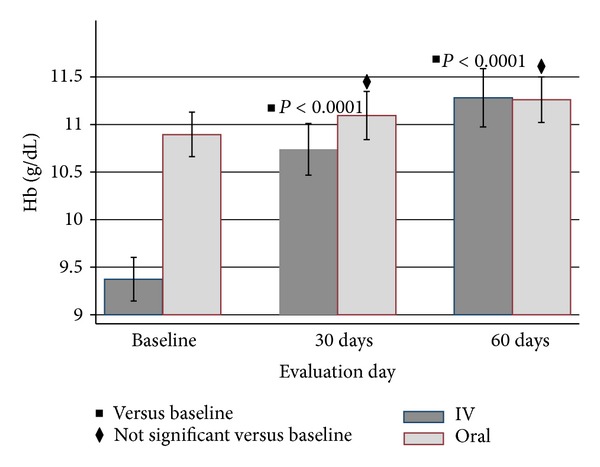
Mean hemoglobin levels ± SD by treatment group over duration of study. *P* values compare 30 and 60 days to baseline values for the IV group.

**Figure 3:**
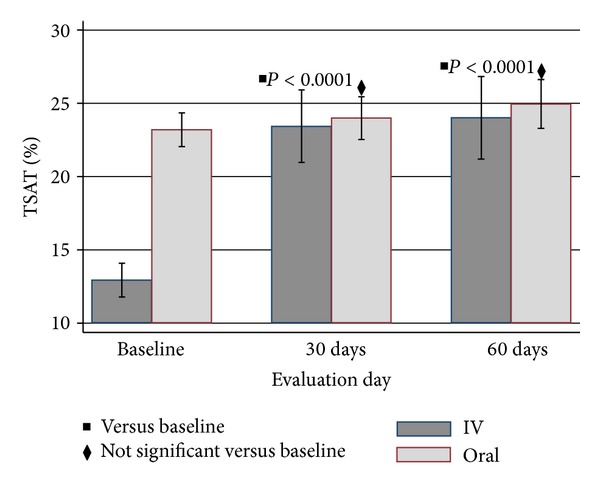
Mean transferrin saturation (TSAT) ± SD levels by treatment group over duration of study. *P* values compare 30 and 60 days to baseline values for the IV group.

**Figure 4:**
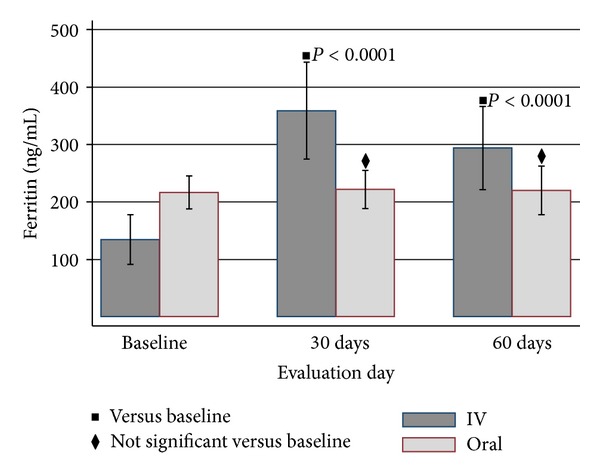
Mean ferritin ± SD by treatment group over duration of study. *P* values compare 30 and 60 days to baseline values for the IV group.

**Figure 5:**
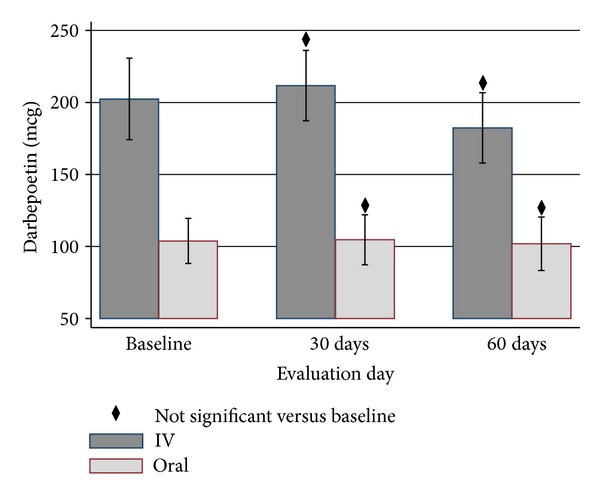
Darbepoetin alfa (mcg) ± SD utilization by treatment group over duration of study.

Figures 2, 3, 4, and 5 show the mean Hb, TSAT, ferritin, and DA levels, respectively, over time by treatment group. Tables 2 and 3 display pairwise comparison of these parameters at three evaluation days in the iron dextran group (IDG) and oral group (OG), respectively. The IDG showed significant improvement in the mean Hb compared to baseline at days 30 (10.7 versus 9.4 g/dL; *P* < 0.0001) and 60 (11.3 versus 9.4 g/dL; *P* < 0.0001). In the OG, mean Hb also increased at 30 days (11.1 versus 10.9 g/dL; *P* = 0.0258) and 60 days (11.3 versus 10.9 g/dL; *P* = 0.0003).

TSAT in IDG improved significantly by day 30 (23.4 versus 12.9; *P* < 0.0001). The effect of 1 gram ID on TSAT was maintained through day 60 (24.0 versus 12.9; *P* < 0.0001). The mean TSAT was maintained by oral iron and increased slightly from baseline (25.0 versus 23.2; *P* = 0.0401). A robust increase in ferritin from baseline was observed in the IDG at day 30 (359.0 versus 134.7; *P* < 0.001). The iron stores as reflected by ferritin remained higher than baseline at day 60 (294.0 versus 134.7; *P* < 0.001). No statistically significant change in ferritin was seen in the OG by 60 days (220.2 versus 216.7; *P* = 0.8339). DA use, as prescribed by the algorithm, was greater in IDG than OG at all evaluation days (*P* < 0.001) as shown in Figure 5. By day 60, Hb, TSAT, and ferritin were not statistically different between the IDG and OG (Hb: 11.3 ± 1.1 versus 11.3 ± 1.2; TSAT: 24.0 ± 9.9 versus 25.0 ± 8.4; ferritin: 294.0 ± 254.6 versus 220.2 ± 213.5; all *P* values > 0.20).

## 4. Discussion

### 4.1. Safety

Four main iron preparations are used in the USA: two iron dextrans (LMWID, INFeD; HMWID, Dexferrum), sodium ferric gluconate (Ferrlecit), iron sucrose (Venofer), and ferumoxytol (Feraheme) [23–27]. Both INFeD and Dexferrum are FDA approved for patients with documented iron deficiency in whom oral administration was unsatisfactory or not possible. In addition to hemodialysis patients [1, 28, 29], the efficacy of iron dextran has been demonstrated in different patient populations with documented iron deficiency, such as patients with pregnancy-related anemia [30], cancer patients [31], surgical patients who refuse transfusions [32], and patients on ambulatory peritoneal dialysis [33, 34]. Among the iron preparations used in the USA, the iron dextrans can be administered as TDI [14, 34–36]. The accelerated TDI of iron dextran was also recently reported safe by Auerbach et al. [37]. Slow infusion of high-dose ID (0.5–1.0 g) is frequently termed TDI because it provides enough iron to produce the desired Hb increase as well as to replete tissue and reticuloendothelial stores. TDI is deemed appropriate by the National Kidney Foundation's Kidney Disease Outcomes Quality Initiative guidelines in CKD patients who despite oral iron supplementation have persistent evidence of iron deficiency [38]. However, TDI is not FDA approved because of high incidence of delayed arthralgias and myalgias [39]. These symptoms usually occur about 24 hours later, abate without therapy, and should not be considered ADEs. Both the HMWID and LMWID carry a black box warning regarding the risk of anaphylactoid reactions. Although anaphylaxis has been reported with LMWID, its incidence is rare (<1 : 200 000). Acute chest and back pain at times are mistakenly described as anaphylaxis attributable to LMWID. These symptoms when not accompanied by hypotension, tachypnea, tachycardia, wheezing, stridor, or orbital edema are harmless, abate in few minutes, and do not automatically qualify for treatment by diphenhydramine or epinephrine. Further premedication with diphenhydramine can cause tachycardia, sweating, somnolence, supraventricular tachycardia, and hypotension, all of which are attributed to the IV iron [40]. Prior to initiating IV iron dextran therapy, the package insert states “administer a test INFeD dose prior to the first therapeutic dose. If no signs or symptoms of anaphylactic-type reactions follow the test dose, administer the full therapeutic INFeD dose” [23].

Criticism and controversy surrounding iron dextran's side effect profile surfaced in its postmarketing phase. Although US sales for iron preparations have soared since the late 1990s, there has been a noticeable decline in ID use. This decline was likely secondary to the market's introduction of newer IV formulations and at least partially due to the adverse events reported in several studies [15, 41–43]. However, none of these observational studies could establish whether other iron formulations possess a superior adverse effect profile than LMWID. Moreover, in 2010, the FDA issued a warning letter to Luitpold Pharmaceuticals to stop claiming a safety advantage over iron dextrans in the absence of comparative clinical trials (pers comm, M Safarik, Department of Health and Human Services, FDA). Hamstra et al. administered 2099 IV injections of iron dextran (Imferon) to 481 patients. Three life-threatening, immediate anaphylactoid and eight severe, delayed reactions were observed [15]. Bailie et al. compared ADEs among three iron preparations (iron dextran, Ferrlecit, and Venofer) using data from the Freedom of Information surveillance database from January 1997 to September 2002. The iron formulations were administered and reported per 100 mg dose equivalents. The all-event reporting rates for iron dextran, sodium ferric gluconate, and iron sucrose were 29.2, 10.5, and 4.2 reports per million 100 mg dose equivalents, while the all-fatal-event reporting rates were 1.4, 0.6, and 0.0 reports per million 100 mg dose equivalents [41]. This paper did not distinguish between HMWID and LMWID formulations, nor did it characterize the population being studied. It estimated ADEs per dose of iron administered. Faich and Strobos reviewed the spontaneous adverse reaction reporting of iron dextran in the USA and sodium ferric gluconate in Germany and Italy from 1976 to 1996. Data was gathered from the World Health Organization, pharmaceutical manufacturers, and the German Health Bureau. The 21-year period had 74 reports of suspected allergic or anaphylactoid reactions for sodium ferric gluconate complex and 196 comparable reports for iron dextran in the US. No fatalities were reported for iron gluconate. However, 31 fatalities were reported for iron dextran [42]. The comparison was made based on the assumption that the extent of drug exposure to both IV preparations was similar during that time period. Data for iron dextrans was combined because reporting databases did not distinguish between the different iron dextran formulations. Fishbane conducted a retrospective chart review of 573 hemodialysis patients treated with IV iron dextran (INFeD) over a 2-year period. Twenty-seven patients (4.7%) had adverse reactions. Four patients (0.7%) had reactions classified as serious [43]. Although the ADE rate appears high in this study, the unit of evaluation was neither the dose nor the episode of IV iron dextran administration but the patient.

Clinicians should be aware that LMWID and HMWID are not clinically interchangeable. Several publications have demonstrated higher rates of adverse events with HMWID [17–20]. Chertow and colleagues examined ADEs among three iron formulations (HMWID, LMWID, and sodium ferric gluconate) using data from the FDA during 1998–2000. The total number of reported parenteral iron-related ADEs was 1981 among approximately 21 060 000 doses. The total major ADE rates were significantly higher among recipients of HMWID and sodium ferric gluconate complex than among recipients of LMWID [17].

In 2006, Chertow et al. examined ADEs among four iron formulations (HMWID, LMWID, sodium ferric gluconate complex, and iron sucrose) using FDA MedWatch data from 2001 to 2003. The rates of both total and life-threatening ADEs were significantly higher among recipientsof HMWID than LMWID or other iron formulations. The total ADE reporting rates for Dexferrum, INFeD, sodium ferric gluconate, and iron sucrose were 129, 40.2, 19.4, and 19.8 reports per million/100 mg equivalents, respectively, whereas the absolute rates of life-threatening events were 11.3, 3.3, 0.6, and 0.9 per million. The investigators point out that several life-threatening ADEs did not specify the iron dextran formulation but were attributed to LMWID, without definitive data. However, when HMWID was avoided, the other formulations were safe with an estimated serious adverse event rate of <1 : 200,000 [18]. In 2001, Fletes et al. reported on iron dextran-related ADEs using data from Fresenius Medical Care North America (FMCNA) clinical variance reports. Among 841 252 IV iron dextran administrations from October 1998 through March 1999 in patients with end-stage renal disease, there were 165 reported suspected ADEs, corresponding to an overall rate of 0.000196%, or approximately 20 per 100 000 doses. Eighteen patients required hospitalization and one patient died. Dyspnea, hypotension, and neurological symptoms were the most common major ADEs. ADEs were 8.1-fold more common among patients administered Dexferrum. The paper did not compare the severity of ADEs between the two iron formulations. Patients with ADEs had a mean age of 61.7 years; almost 50% of ADEs occurred in women; 66% occurred on initial dosing and with a median time of 5 minutes into dosing [19].

In our study, all ADEs occurred in older women (mean age 70.3 years), on initial dosing, and within 30 minutes of dosing. None of our patients required emergency evaluation, was hospitalized, or had fatal outcomes. While the retrospective nature of this study would likely underestimate minor reactions, it likely offers a reasonable estimation of major adverse events. Our major ADE rate was 1 per 1699 total iron dextran infusions. Our major ADE rate per 100 mg dose would translate to 12 per 100,000. However, we strongly feel that ADEs reported per episode of IV iron administered better reflect the true event rate. The study also shows that slow infusion of high-dose INFeD is well tolerated in ND-CKD patients. Unlike the nondextran IV formulations where repeated IV infusions are required to achieve iron repletion, it can be conveniently administered in a single clinic visit.

### 4.2. Efficacy

Efficacy studies of IV iron undertaken in predialysis chronic renal failure have yielded contradictory results. Some randomized controlled trials have shown that IV iron is superior to oral iron in raising Hb in ND-CKD patients [44–46] whereas other trials failed to demonstrate the same [47–49]. Only one trial [47] was conducted exclusively in non-ESA-treated patients. Aggarwal et al. [44] randomized 40 patients to either oral ferrous sulfate, 200 mg three times a day (TID), or iron dextran, 100 mg IV (Imferon) twice a month, and followed for up to 3 months. All patients were given ESA therapy. IV iron dextran was more effective than oral iron sulfate in increasing Hb and in improving iron parameters. Van Wyck et al. [45] randomized 188 ESA-treated and non-ESA-treated patients to either oral ferrous sulfate, 325 mg for 56 days, or iron sucrose, 1 g IV in divided doses over a 2-week period. IV iron sucrose was more effective than oral ferrous sulfate in improving iron indices and in increasing Hb levels by at least 1 g/dL at any time point during the study. Spinowitz et al. [46] randomized 304 ESA-treated and non-ESA-treated patients to either two 510 mg of ferumoxytol or 200 mg of elemental iron for 21 days. At day 35, ferumoxytol significantly increased iron indices and hemoglobin compared to oral iron.

Charytan et al. [48] randomized 96 patients to either oral iron for 29 days or iron sucrose 200 mg IV once a week for 5 doses and followed patients for 43 days. No significant difference was found between the two groups in Hb levels, but serum ferritin levels improved more in the IV iron sucrose group. Stoves et al. [49] randomized 45 patients to either oral ferrous sulfate, 200 mg TID, or iron sucrose, 300 mg IV once a month, and followed them for an average of 5.2 months. All patients were simultaneously started on ESA, and the dose was adjusted according to a preestablished protocol. Although serum ferritin improved more in the IV group, iron sucrose was not superior to ferrous sulfate in improving Hb or decreasing the ESA doses. Agarwal et al. [47] showed that ferrous sulfate, 325 mg three times a day × 42 days, and sodium ferric gluconate, 250 mg IV weekly × 4, similarly increase Hb in anemic iron-depleted ND-CKD patients not receiving ESAs.

The aim of our study was to demonstrate the efficacy of the computerized anemia management protocol. We used IV iron in the form of LMWID. ID allows for high-dose infusions, giving it a practical advantage over other IV formulations. The marketed “safer preparations” require smaller, more frequent infusions over a longer period of time. These tend to be more costly in terms of IV tubing, monitoring, IV access, nursing, and allotted time. In theory, at least, repeated injury to the venous system poses potential problems with future venous access. In our protocol, in the group assigned to LMWID + ESA, Hb increased by 1.3 ± 1 g/dL and 1.9 ± 1 g/dL at days 30 and 60, respectively. Despite the robust rise in Hb in the IDG and the incorporation of transferrin bound iron to erythroid precursors, TSAT increased considerably at days 30 (23.4 versus 12.9; *P* < 0.0001) and 60 (24.0 versus 12.9; *P* < 0.0001). Iron stores as reflected by ferritin increased significantly from baseline at day 30 and remained higher than baseline at day 60. By *≈*60 days, mean Hb, TSAT, and ferritin in IDG all increased to levels dictating transition to oral iron replacement per protocol (Figure 1). Our results support that LMWID when combined with ESA is efficacious in raising Hb and replenishing iron stores in as early as *≈*30 days in ND-CKD patients. It also supports that high-dose LMWID should be considered in ND-CKD patients who fail or cannot tolerate oral iron.

The limitation of this study is its retrospective design which is subject to reporting bias. Adherence to oral iron was not guaranteed. Patients were not segregated into ESA naive and nonnaive. A predominant fraction of the patients in both groups had already been managed in the CKD clinic and had received DA in the three months prior to being enrolled in CAMP.

### 4.3. Costs

Table 4 represents the Average Selling Price (ASP) for different iron formulations based on Centers for Medicare and Medicaid Services for the fourth quarter of 2012. In the USA, the ASP of ferumoxytol is more than all the other formulations. The ASPs of iron sucrose and ferric gluconate are comparable. Both of these drugs have higher ASPs than the iron dextrans.

### 4.4. Prospects for Future

Each of the currently available parenteral iron preparations has safety or convenience limitations in patients not on dialysis. The nondextran formulations available in the USA are not recommended for TDI, and multiple administrations must be scheduled. In Europe, two other formulations are approved for TDI in 15 minutes: ferric carboxymaltose (1000 mg) and iron isomaltoside (20 mg/kg). Ferumoxytol is approved in the USA [37], and there might be emerging data that it could be given in TDI (abstract, Am Soc Hematol 54th Annual Meeting, 2012). However, the characteristics of the ADE make it difficult to conduct comparative safety studies between the different formulations with sufficient methodological consistency and statistical power. Moreover, adequately powered prospective comparative trials between two iron formulations will likely never be done due to the large sample sizes needed. For example, at 90% power, to detect a difference between iron dextran with our reported serious ADE rate (1/1699) and an IV formulation with half as common serious ADEs, about 114,000 patients are required in each group.

## 5. Conclusion

Our study supports the use of LMWID (INFeD) in TDI. It is safe and effective in the treatment of iron deficiency in ND-CKD. Because fatal anaphylactic reactions have been reported after administration of iron dextran injection, the drug should be given only when resuscitation techniques and treatment of anaphylactic and anaphylactoid shock are readily available. Our study did not show that higher doses of ID were associated with worse ADE rates. If other potential formulations are used, ADE profile, potential benefits, and relative costs should be established.

## Figures and Tables

**Table 1:** Patient demographics and baseline characteristics.

Baseline results	IV iron	Oral iron	*P* value
	(*n* = 50)	(*n* = 100)	
Demographics			
Age, years	69.1 ± 12.9	65 ± 13.5	0.061^†^
Race, *n* (White/Black/Asian/Hispanic)	10/40/0/0	14/83/1/2	0.654^#^
Gender, *n* (male/female)	23/27	45/55	1.000^*χ*^
Former medical history, *n*			
Congestive heart failure	18 (36%)	13 (13%)	0.001^*χ*^
Coronary artery disease	12 (24%)	16 (16%)	0.236^*χ*^
Hypertension	49 (98%)	97 (97%)	1.000^#^
Diabetes	25 (50%)	54 (54%)	0.643^*χ*^
Laboratory			
Hb (g/dL)	9.4 ± 0.8	10.9 ± 1.2	<0.001^†^
TSAT%	12.9 ± 4.0	23.2 ± 5.8	<0.001^†^
Ferritin (ng/mL)	134.7 ± 151.5	216.7 ± 146.0	<0.005^†^

^†^2-sample  *t*-test, ^*χ*^Chi-square, ^#^Fisher exact.

Demographics are reported as frequencies except for age which is reported as a mean. Variables under past medical history are reported as frequencies with percentages in parenthesis. Laboratory parameters are reported as means + SD. *P* value > 0.05 was considered nonsignificant (n.s.).

**Table 2:** Parameter comparison at three evaluation points in iron dextran group.

Parameter	Evaluation days	Group means ± SD		*P* value
Hb (g/dL)	Baseline versus day 30	9.4 ± 0.8	10.7 ± 1.0	<0.0001
	Baseline versus day 60	9.4 ± 0.8	11.3 ± 1.1	<0.0001
	Day 30 versus day 60	10.7 ± 1.0	11.3 ± 1.1	<0.0001

TSAT%	Baseline versus day 30	12.9 ± 4.0	23.4 ± 8.7	<0.0001
	Baseline versus day 60	12.9 ± 4.0	24.0 ± 9.9	<0.0001
	Day 30 versus day 60	23.4 ± 8.7	24.0 ± 9.9	<0.0001

	Baseline versus day 30	134.7 ± 151.5	359.0 ± 295.6	<0.0001
Ferritin (ng/mL)	Baseline versus day 60	134.7 ± 151.5	294.0 ± 254.6	<0.0001
	Day 30 versus day 60	359.0 ± 295.6	294.0 ± 254.6	0.0058

**Table 3:** Parameter comparison at three evaluation points in oral iron group.

Parameter	Evaluation days	Group means ± SD		*P* value
Hb (g/dL)	Baseline versus day 30	10.9 ± 1.2	11.1 ± 1.3	0.0258
	Baseline versus day 60	10.9 ± 1.2	11.3 ± 1.2	0.0003
	Day 30 versus day 60	11.1 ± 1.3	11.3 ± 1.2	0.0458

TSAT%	Baseline versus day 30	23.2 ± 5.8	24.0 ± 7.3	0.3318
	Baseline versus day 60	23.2 ± 5.8	25.0 ± 8.4	0.0401
	Day 30 versus day 60	24.0 ± 7.3	25.0 ± 8.4	0.2889

	Baseline versus day 30	216.7 ± 146.0	221.9 ± 167.0	0.4722
Ferritin (ng/mL)	Baseline versus day 60	216.7 ± 146.0	220.2 ± 213.5	0.8339
	Day 30 versus day 60	221.9 ± 167.0	220.2 ± 213.5	0.9215

**Table 4:** Average selling price (ASP) based on Centers for Medicaid and Medicare Services.

Product name	ASP per gram
Iron dextran (Infed)	$240.0
Iron dextran (Dexferrum)	$240.0
Iron sucrose (Venofer)	$285.0
Sodium ferric gluconate (Ferrlecit)	$265.2
Ferumoxytol (Feraheme)	$638.0
